# Novel, Environment-Friendly Cellulose-Based Derivatives for Tetraconazole Removal from Aqueous Solution

**DOI:** 10.3390/polym13030450

**Published:** 2021-01-30

**Authors:** Bayan Khalaf, Othman Hamed, Shehdeh Jodeh, Ghadir Hanbali, Roland Bol, Omar Dagdag, Subhi Samhan

**Affiliations:** 1 Department of Chemistry, Faculty of Science, An-Najah National University, P.O. Box 7, Nablus, Palestine; bayan.kh107@hotmail.com (B.K.); g.hanbali@najah.edu (G.H.); 2 Institute of Bio and Geosciences, Agrosphere (IBG-3), Forschungszentrum Jülich GmbH, 52425 Jülich, Germany; r.bol@fz-juelich.de; 3 Laboratory of Industrial Technologies and Services (LITS), Department of Process Engineering, Height School of Technology, Sidi Mohammed Ben Abdallah University, P.O. Box 2427, 30000 Fez, Morocco; omar.dagdag@uit.ac.ma; 4 Research and Development Center, Palestine Water Authority, Ramallah 00001, Palestine; subhisamhan@yahoo.com

**Keywords:** cellulose, pesticides, tetraconazole, furan-2-carbonyl chloride, pyridine-2,6-dicarbonyl dichloride, water purification

## Abstract

In this study, cellulose-based derivatives with heterocyclic moieties were synthesized by reacting cellulose with furan-2-carbonyl chloride (Cell-F) and pyridine-2,6-dicarbonyl dichloride (Cell-P). The derivatives were evaluated as adsorbents for the pesticide tetraconazole from aqueous solution. The prepared adsorbents were characterized by SEM, TGA, IR, and H^1^ NMR instruments. To maximize the adsorption efficiency of tetraconazole, the optimum conditions of contact time, pH, temperature, adsorbent dose, and initial concentration of adsorbate were determined. The highest removal percentage of tetraconazole from water was 98.51% and 95% using Cell-F and Cell-P, respectively. Underivatized nanocellulose was also evaluated as an adsorbent for tetraconazole for comparison purpose, and it showed a removal efficiency of about 91.73%. The best equilibrium adsorption isotherm model of each process was investigated based on the experimental and calculated *R*^2^ values of Freundlich and Langmuir models. The adsorption kinetics were also investigated using pseudo-first-order, pseudo-second-order, and intra-particle-diffusion adsorption kinetic models. The Van’t Hoff plot was also studied for each adsorption to determine the changes in adsorption enthalpy (∆*H*), Gibbs free energy (∆*G*), and entropy (∆*S*). The obtained results showed that adsorption by Cell-F and Cell-P follow the Langmuir adsorption isotherm and the mechanism follows the pseudo-second-order kinetic adsorption model. The obtained negative values of the thermodynamic parameter ∆*G* (−4.693, −4.792, −5.549 kJ) for nanocellulose, Cell-F, and Cell-P, respectively, indicate a spontaneous adsorption process. Cell-F and Cell-P could be promising absorbents on a commercial scale for tetraconazole and other pesticides.

## 1. Introduction

The large use of pesticides is a global concern due to their ability to biomagnify and bioaccumulate in the ecosystems, in addition to their significant adverse effects on both humankind and the environment [1,2,3]. Several methods for removal of pesticides including tetraconazole from water have been developed, including ultrasonication, membrane technology, UV photolysis, Fenton oxidation, adsorption, electrochemical degradation, photocatalyst, and several others. In comparison with other treatment techniques, the adsorption method for pesticides shows obvious advantages, including a lower cost, ease of operation, and reusability [3,4,5,6,7,8,9,10,11,12]. Previous studies have also suggested the removal of tetraconazole by using mesoporous alumina and light-induced degradation [13,14].

Among the pesticides that are widely used, with their concentrations in soil and water piled up to a harmful level, is tetraconazole, which is a fungicide of the triazole group. It is effective in controlling a broad spectrum of diseases, for example, powdery mildew on vines and cucumbers and rust on vegetables [15]. Hydrolysis and photolysis of tetraconazole proceed slowly in soil such that laboratory and field studies estimate the half-time dependent on its application, soil texture, and concentration in the range of 69 days to over 1688 days [15,16].

Tetraconazole can behave like either a lipophilic or a hydrophilic compound due to the existence of a tetrafluoroethoxy group. Its well-balanced hydrosolubility/liposolubility ratio results in perfect systemic activity [15]. This pesticide can readily enter the target plants and distributes equally throughout all the treated plant tissues, which results in a high level of protection, even in the untreated sections or in the vegetation grown after spraying. The bioaccumulation and persistency of tetraconazole adversely affects humankind and the environment; for example, it can cause kidney failure, liver damage, and nervous system disorder [15,16].

Cellulose is considered one of the most abundant renewable and biological materials in the world. It has been industrial feedstock for a large number of derivatives with an unlimited number of commercial applications. Surface-modified cellulose can also be of great interest due to a wide range of applications [17,18,19,20,21,22,23,24]. This work aims to purify water from tetraconazole using a novel synthesized cellulosic polymer modified with furan-2-carbonyl chloride (Cell-F) and crosslinked with pyridine-2,6-dicarbonyl dichloride (Cell-P). The efficiency of the prepared cellulose polymer toward tetraconazole was investigated as a function of adsorbent dose, temperature, pH, and time. In addition, isotherms, kinetics, and thermodynamic parameters were determined. The novelty of this work can be summarized as being, to the best of our knowledge, the first examples in the literature of removing tetraconazole from water.

## 2. Experimental

### 2.1. Materials

All chemicals used in this work were of analytical grade. They were purchased from Sigma-Aldrich Chemical Company (Munich, Germany) and used without any further purification, unless otherwise specified.

The chemicals used in this work include dimethyl sulfoxide, furan-2-carbonyl chloride, pyridine-2,6-dicarbonyl dichloride, anhydrous lithium chloride, methanol, triethylamine, anhydrous dimethylacetamide, sulfuric acid, acetonitrile, and tetraconazole.

### 2.2. Methods

^1^H NMR spectra were recorded at Forschungszentrum Jülich, Jülich, Germany, using a Bruker 600 MHz spectrometer equipped with a 5 mm broadband CryoProbe Prodigy. The acquisition parameters were as follow: 90° pulse calibrated at 12 μs, 1.3 s acquisition time, 2 s relaxation time, no spinning, 300 K, and 2048 scans. Ultraviolet–visible spectroscopy was recorded using UV-1601 (SHIMADZU), and an IR spectrometer (Nicolet iS5, iD3 ATR, Thermo Scientific, Japan) was used in this work. In addition, thermogravimetric analysis was performed using a Q50 V20.10 TGA, Build 36 instrument at a heating rate of 10 °C/min in N_2_ gaseous atmosphere (New Castle, DE, USA). UV-Visible spectroscopy, IR and TGA analysis were obtained at An-Najah National University, Nablus, Palestine.

To study the adsorbents’ surface morphology, we used scanning electron microscopy (SEM). For this, the samples were fixed onto a double-sided C tape and mounted onto a sample holder. All samples were subjected to sputter-coating of a 10-nm-thick gold film onto them in order to minimize charging effects for taking high-magnification images. The samples were placed into a vacuum sample chamber, and micrographs were obtained using an emission scanning electron microscope (JEOL 7400F; Oxford Instruments Inca, Tubney Woods, Abingdon, Oxon OX13 5QX, UK).

High- performance liquid chromatography (HPLC) analysis was performed at Nablus, Palestine. HPLC was obtained using a mobile phase degasser unit (Model: DGU-20A3), 30 µL sample loops, pump (Model: LC-20AB), and a photo diode array detector (PDD) (Model: SPD-M20A), manufactured by Shimadzu Corporation, Japan. The data were analyzed using Breeze QS software. The HPLC instrument was equipped with an X TERRA C18 column (5 μm, 250 mm × 4.6 mm). The mobile phase comprised 64% acetonitrile and 36% distilled water. The analysis was carried out at 220 nm wavelength. The flow rate was 1 mL/min.

### 2.3. Preparation of Cellulose Nanocrystalline

Cellulose (5.0 g) was added to a beaker (500 mL) containing distilled water (200 mL) and stirred magnetically for 30 min. Then, 20.0 g of concentrated H_2_SO_4_ was added to produce a solution with 10 wt% of sulfuric acid. The mixture was stirred at 60 °C for 2 h. The hot mixture was then diluted with ice-cold distilled water. The colloidal suspension was centrifuged at 10,000 rpm and decanted. The process was repeated three times to remove the hydrolysis product and H_2_SO_4_.

### 2.4. Cellulose Dissolution

The dissolution of cellulose was carried out by activation of cellulose (1.0 g), first in water (100 mL) at room temperature, where the suspension was stirred for 2 h, followed by suction filtration and suspension of the collected cellulose for 1 h in 100 mL of methanol. The procedure was repeated twice, followed by two successive exchanges of 25 mL volume of anhydrous *N,N*-dimethylacetamide (DMAc). The first DMAc exchange was done for 1 h, while the second one was carried out for one night. After each solvent exchange, DMAc was removed using filtration under vacuum by a glass-centered funnel. The activated cellulose was then transferred to a two-necked round-bottomed flask equipped with a condenser and a magnetic stir bar. After that, the flask was connected to a poplar via the condenser. A solution of 6.5 g of anhydrous lithium chloride (LiCl) in 100 mL of anhydrous *N,N*-dimethylacetamide (DMAc) was added to the flask. The mixture was then stirred for 2 h until a clear gel was obtained.

### 2.5. Cellulose Acylation with Furan-2-Carbonyl Chloride

To the cellulose solution in LiCl/DMAc prepared before was added trimethylamine (0.5 mL) dropwise under N_2_, followed by a solution of furan-2-carbonyl chloride (2.415 g, 0.0185 mol) in 10 mL of DMAc. The produced reaction mixture was stirred for 3.0 h at 70 °C. It was then transferred to a beaker containing 500 mL of distilled water, and the produced precipitate was collected by suction filtration, washed several times with distilled water and with isopropyl alcohol, and then dried at room temperature.

### 2.6. Cellulose Crosslinking with Pyridine-2,6-Dicarbonyl Dichloride

The above procedure was repeated exactly, except that pyridine-2,6-dicarbonyl dichloride (3.775 g in 10 mL of DMAc) was added to the solution of cellulose in LiCl/DMAc. A precipitate of crosslinked cellulose started appearing after about 30 min from heating the reaction mixture at 70 °C. The produced precipitate was collected by suction filtration, washed several times with distilled water and with ethanol, and then dried at room temperature.

### 2.7. Adsorption Study of Tetraconazole

All experiments were performed in 50 mL plastic vials that were placed in a shaker immersed in a water bath equipped with a thermostat and a shaker. The effect of various variables on polymer efficiency, such as initial concentration (*C*_0_), pH, adsorbent dosage, adsorption time, and temperature, were evaluated. After each run, a sample was collected from the mixture using a 10 mL plastic syringe, filtered through a 0.45 µm filter, and subjected to analysis by HPLC at 220 nm wavelength to determine the residual concentration of tetraconazole. All adsorption experiments were performed in triplicate, and the mean of the three runs was reported. The amount of tetraconazole adsorbed by Cell-F and Cell-P was determined according to Equations (1) and (2), respectively:

(1) $$R\;\left(\%\right)=\;\frac{C_{0}\;-\;C_{e}}{C_{0}}$$

(2) $$Q_{e}=\;\frac{C_{0\;}-\;C_{e}}{m}V$$

where *C*_0_ and *C_e_* are the initial and equilibrium concentrations (ppm) of tetraconazole in solution, respectively; *Q_e_* is the equilibrium adsorption capacity (ppm); *m* is the mass of Cell-F and Cell-P (mg); and *V* is the volume of the solution (L).

## 3. Results and Discussion

### 3.1. Polymer Analysis

**Scheme 1:**
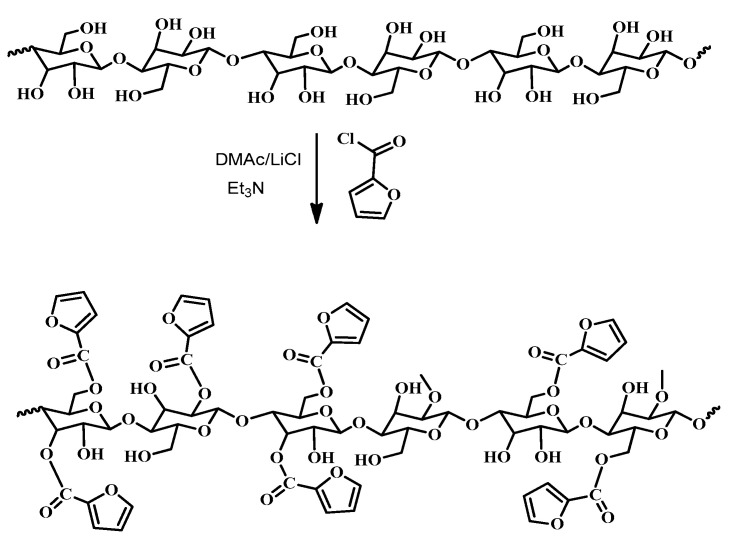
Cellulose acylation with furan-2-carbonyl chloride.

**Scheme 2:**
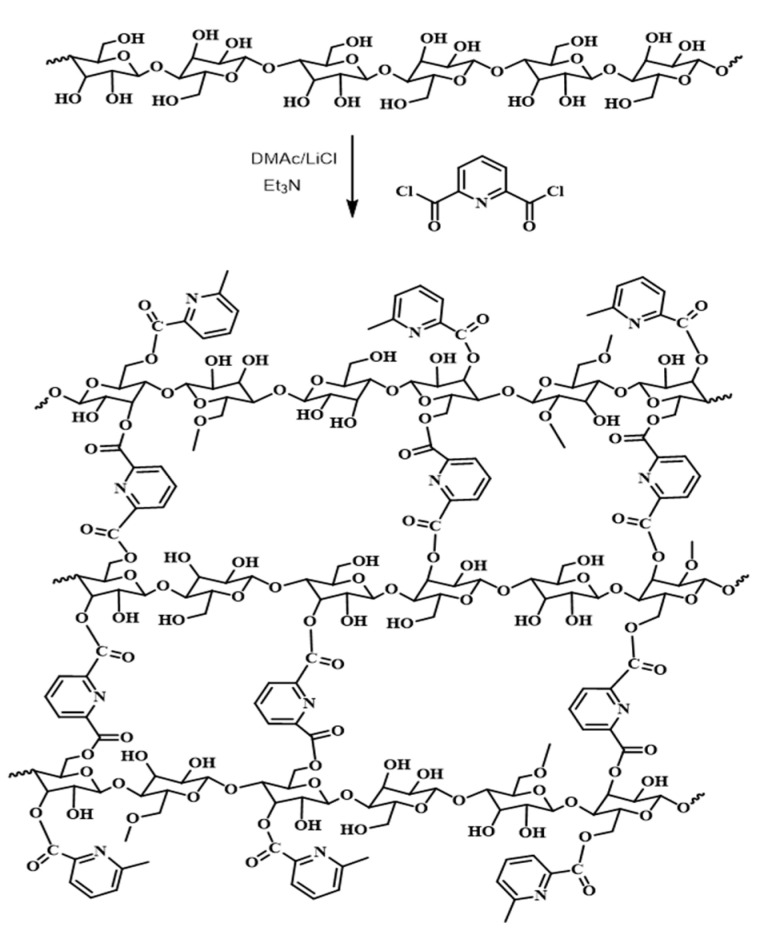
Cellulose crosslinking with pyridine-2,6-dicarbonyl dichloride.

Grafting of cellulose with furan-2-carbonyl group and crosslinking with pyridine-2,6-dicarbonyl dichloride was carried in DMAc/LiCl, as shown in Scheme 1 and Scheme 2. The reagents were chosen for the functional groups they have, since the presence of the aromatic heterocyclic group, the carbonyl group, and the hydroxyl groups of cellulose make them novel adsorbents for tetraconazole. As mentioned before, these functional groups bind with tetraconazole through various physical interactions, including H-bonding, π–π stacking, and dipole–dipole interaction.

#### 3.1.1. InFrared Analysis

The prepared cellulose derivatives, Cell-F and Cell-P, were characterized by FTIR. Cell-F (Figures S1 and S2) showed a band at 1717.61 cm^−1^, which could be attributed to the ester carbonyl, and the low frequency of the carbonyl groups could be due to the conjugation with a heterocyclic double bond. The C=C of the furan ring showed a small peak at 1536 cm^−1^. The band at 1179.74 cm^−1^ corresponds to the C–O–C of cellulose (β-glycosidic linkage). The IR spectrum of Cell-P showed almost similar peaks as Cell-F, with a slight shift in frequency.

#### 3.1.2. Proton Nuclear Magnetic Resonance Analysis

**Figure 1:**
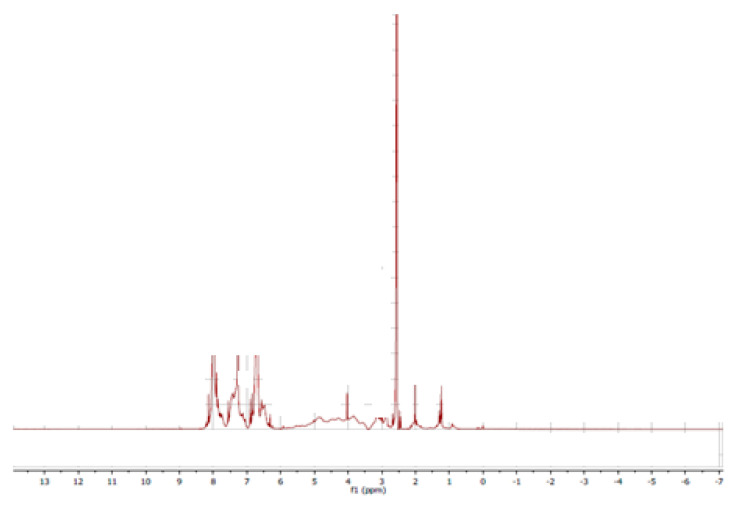
H^1^ NMR spectra of the cellulose nanocrystal (CNC) and furan-2-carbonyl chloride (Cell-F).

The two polymers, in addition to the starting cellulose nanocrystal (CNC), were also characterized by proton NMR spectroscopy. The ^1^H NMR spectrum of the CNC is shown in Figure 1.

A cellulose solution in DMAc-d6/LiCl was obtained by solvent exchange, as shown in the experimental part. The spectrum showed downfield peaks at δ 4.35, which could be assigned to the proton H1 at the anomeric carbon [25]. The two multiple peaks at δ 3.92 and 3.85 could be assigned to the 2Hs at C-6. The results reported related to H6 are consistent with the previous literature [26]. The four peaks between δ 3.1 and 3.60 are from the four C2, C3, C4, and C5 protons. The peak at δ 3.1 is consistent with the chemical shift of H2 [25].

The proton NMR spectrum of Cell-F is shown in Figure 1. The spectrum shows three peaks between 6.71 and 8.12 ppm, which could be attributed to the aromatic furan protons, and the cellulose peaks appear between 2.7 and 5.0 ppm.

We were not able to generate a proton NMR spectrum of Cell-P due to a solubility issue, which could be attributed to crosslinking.

#### 3.1.3. Scanning Electron Microscope (SEM) Analysis

##### SEM Characterization Was Obtained for the Adsorbents CNC, Cell-F and Cell-P

**Figure 2:**
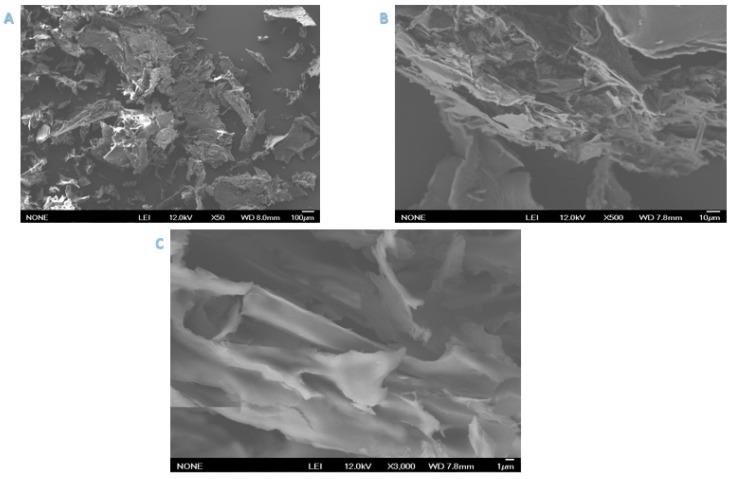
SEM images of the CNC at three different magnifications: (**A**) 100 µm, (**B**) 10 µm, and (**C**) 1 µm.

The obtained SEM images of nanocellulose are shown in Figure 2. Three images were obtained at three different magnifications (1, 10, and 100 µm). The adsorption efficiency is expected to increase as a result of its high surface-to-volume ratio.

**Figure 3:**
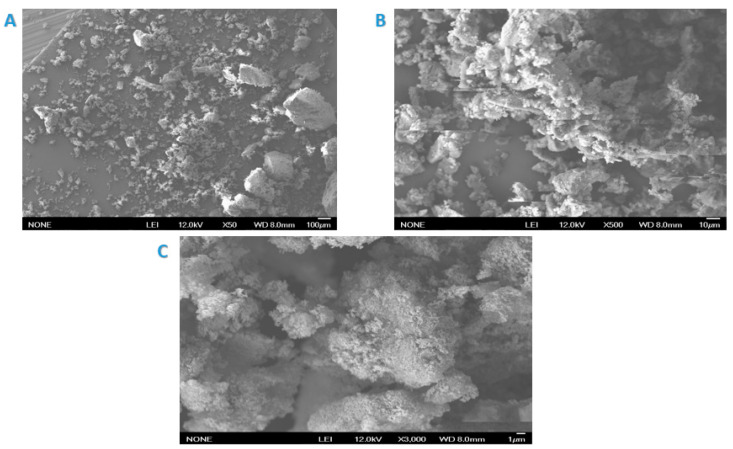
SEM images of Cell-F at three different magnifications: (**A**) 100 µm, (**B**) 10 µm, and (**C**) 1 µm.

The obtained SEM images of Cell-F are shown in Figure 3. Three images were obtained at three different magnifications (1, 10, and 100 µm). As shown below, the polymer is highly porous, with a high surface-to-volume ratio, which enhances the adsorption efficiency of the polymer.

**Figure 4:**
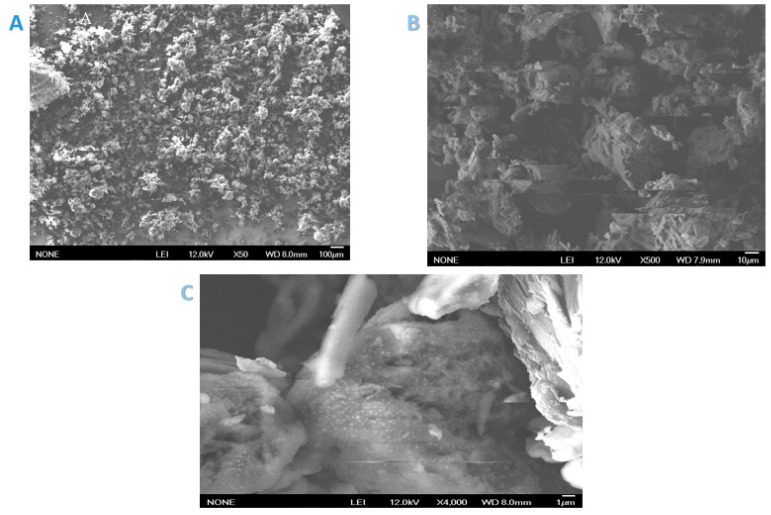
SEM images of pyridine-2,6-dicarbonyl dichloride (Cell-P) at three different magnifications: (**A**) 100 µm, (**B**) 10 µm, and (**C**) 1 µm.

In addition, three images at different magnifications were obtained for Cell-P, as shown in Figure 4, at 1, 10, and 100 µm. Cell-P showed almost a similar morphology as that showed by Cell-F.

#### 3.1.4. Thermogravimetric Analysis (TGA)

**Figure 5:**
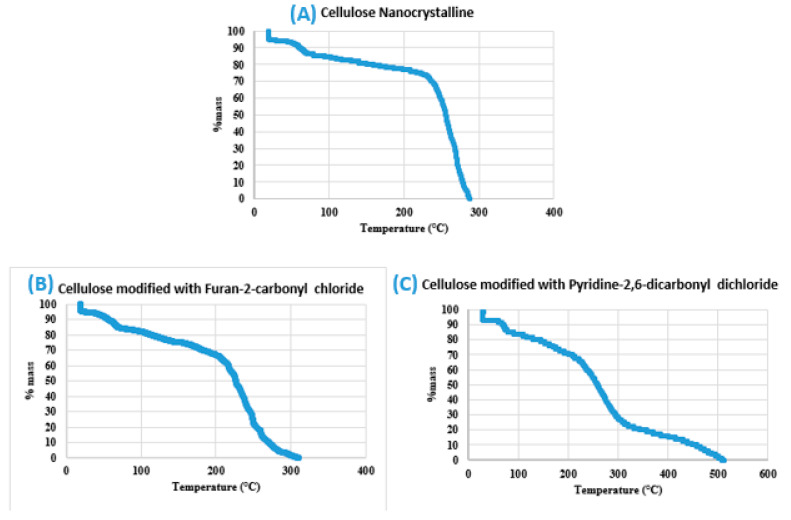
TGA analysis results for (**A**) Cellulose nanocrystal, (**B**) Cellulose modified with Furan-2-carbonyl chloride (Cell-F), (**C**) Cellulose modified with Pyridine-2,6-dicarbonyl dichloride (Cell-P).

TGA was performed on the CNC, Cell-F, and Cell-P, and the results are summarized in Figure 5. The graphs show mass loss as a function of temperature. The three polymers showed stability at a temperature over 200 °C, since the major loss in weight appeared at about 250 °C.

### 3.2. Adsorption Results

#### 3.2.1. Effect of Contact Time

**Figure 6:**
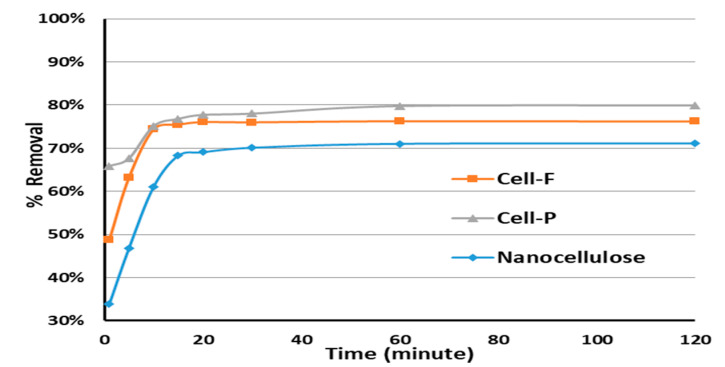
Contact time effect on tetraconazole removal by nanocellulose, Cell-F, and Cell-P (initial concentration (*C*_0_) = 10 ppm, adsorbent dose = 10 mg, solution volume = 10 mL, pH = 7, and temperature = 20 °C).

Adsorption of tetraconazole from water was carried out using the three polymers CNC, Cell-F, and Cell P. The adsorption was evaluated as a function of time at a constant pH (neutral) with initial concentration = 10 ppm, adsorbent dose = 10.0 mg, volume of solution = 10 mL, and temperature = 20 °C. The results are shown in Figure 6. As shown in Figure 6, adsorption started rapidly and almost reached a plateau after 20 min for the three polymers. This could be related to the availability of the binding sites at the beginning, which then became saturated with time. Based on these results, a contact time of 20 min was chosen as the optimal contact time.

This figure also shows that the highest percentage of tetraconazole removal was shown by Cell-P. The percentage of removal reached about 77.8%, 75.5%, and 70.1% for Cell-F, Cell-P, and the CNC, respectively. Cell-P is a crosslinked cellulose (Figure 3) forming a cyclic cavity that is packed with binding sites, which makes Cell-P behave as a unique adsorbent with the ability to trap tetraconazole via various intermolecular forces such as π–π interaction and H-bonding.

#### 3.2.2. Effect of pH

**Figure 7:**
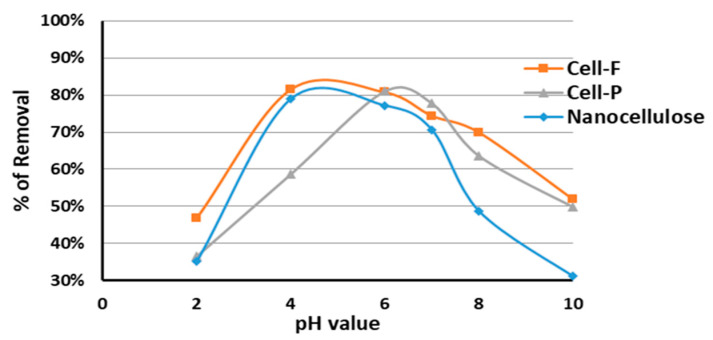
pH effect on tetraconazole removal by nanocellulose, Cell-F, and Cell-P (*C*_I_ = 10 ppm, time = 20 min, adsorbent dose = 10 mg, solution volume = 10 mL, and temperature = 20 °C).

The adsorption efficiency as a function of the pH value is shown in Figure 7; the other variables were kept constant. The pH value is a critical element in adsorption because the pH value can control the surface charge of the adsorbent and adsorbate. At pH lower than 3.0, all tetraconazole amines are in ammonium form (-NR_3_H^+^). Because of this, the adsorption efficiency was very low (less than 40%). However, at pH values higher than 4.0, the amines are in the basic form, and a lone pair of electrons are available on the N for binding. The highest adsorption efficiency was observed at a pH value in the range of 4.0 to 7.0.

This highest removal was about 81.5% for both Cell-F and Cell-P. The cellulose nanocrystal showed a lower efficiency, and the percentage removal was about 79%.

#### 3.2.3. Effect of Tetraconazole Concentration

**Figure 8:**
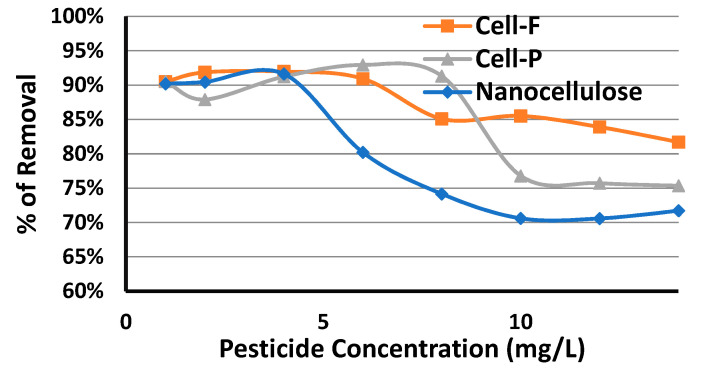
Pesticide concentration effect on tetraconazole removal by nanocellulose, Cell-F, and Cell-P (adsorbent dose = 10 mg, solution volume = 10 mL, and temperature = 20 °C).

The effect of the initial concentration of tetraconazole on the percentage removal using the three adsorbents was investigated, the other variables being kept constant (pH = 7, time = 20 min, adsorbent dose = 10 mg, solution volume = 10 mL, temperature = 20 °C). The maximum percentage of tetraconazole removal was about 92.9% for Cell-P at 6 ppm concentration of tetraconazole (Figure 8).

At a low concentration of tetraconazole, the limiting factor for adsorption is the initial concentration and reaches the highest at 4.0 ppm; then as the ion concentration increases, the adsorption decreases. At a concentration higher than 4.0 ppm, the availability of the binding sites becomes the limiting parameter, and they are limited by the amount of adsorbent. Before reaching the optimum tetraconazole concentrations using the CNC, Cell-F, and Cell-P, there were still available sites for binding between the pesticide and the adsorbent. For concentrations higher than 4 ppm, 8 ppm, and 6 ppm for the CNC, Cell-F, and Cell-P, respectively, saturation of the adsorbent at available binding sites takes place.

#### 3.2.4. Effect of Temperature

**Figure 9:**
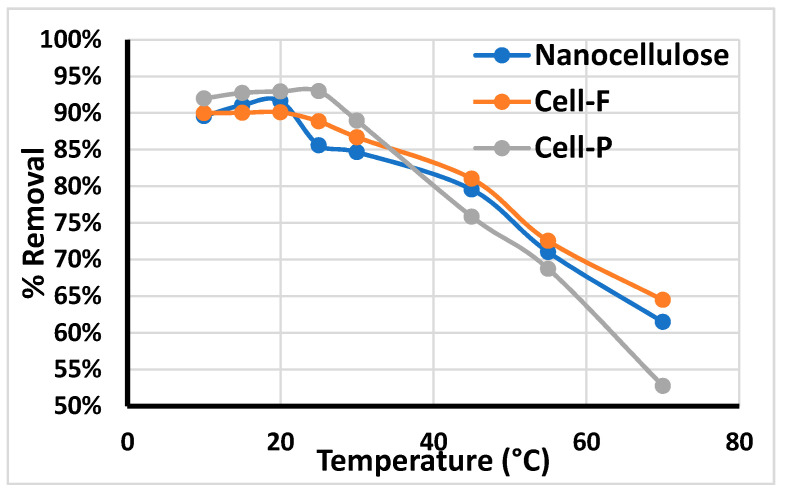
Temperature effect on tetraconazole removal by nanocellulose, Cell-F, and Cell-P (adsorbent dose = 10 mg, solution volume = 10 mL, and pH = 4 for CNC and Cell-F, pH = 6 for Cell-P).

The temperature effect on the adsorption of tetraconazole by the CNC, Cell-F, and Cell-P were also evaluated, the other variables being kept constant, as shown below. The results are summarized in Figure 9. The maximum adsorption efficiency was observed at about 25 °C. At a temperature over 25 °C, the percentage of tetraconazole removal started to decrease, such that heating adversely affected the adsorbents’ thermal stability (Figure 5). 

#### 3.2.5. Effect of Adsorbent Dose

**Figure 10:**
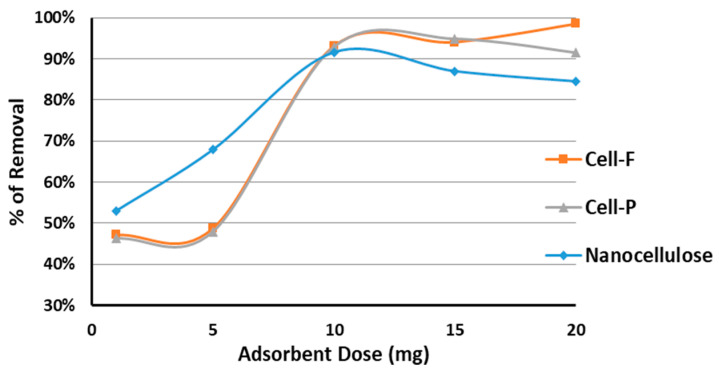
Adsorbent dose effect on tetraconazole removal by nanocellulose, Cell-F, and Cell-P (solution volume = 10 mL).

The polymer dosage that provided the highest adsorption efficiency was selected as the optimal dosage. As shown in Figure 10, the highest percentage of removal was attained using about 12.0 mg of all polymers. The rate of adsorption of tetraconazole increased with increasing polymer dosage. Tetraconazole removal reached approximately 98.7% using Cell-F and Cell-P. The highest percentage of removal by the CNC was about 91.7%. The results indicated that the adsorption process could be controlled by diffusion and surface coordination mechanisms. An increase in the polymer dosage resulted in an increase in the number of available adsorption sites; as a result, the rate of tetraconazole adsorption increased. When all adsorption sites get occupied, the diffusion process begins, which is controlled by osmosis, so as the concentration of the adsorbate in the polymer body becomes equal to that in the solution, the adsorption reaches a plateau.

As shown in Figure 10, the optimum doses were 10 mg, 20 mg, and 15 mg for the CNC, Cell-F, and Cell-P, respectively. The lower optimum mass of nanocellulose was due to its high surface-to-volume ratio. However, the effect of the percentage removal of tetraconazole at doses greater than 10 mg for all polymers was low.

### 3.3. Adsorption Analysis

The best adsorption isotherm was investigated depending on the closeness of the correlation coefficient (*R*^2^) to both Freundlich and Langmuir models. Adsorption kinetic models were also determined using pseudo-first-order, pseudo-second-order, and intra-particle-diffusion adsorption models. A Van’t Hoff graph was investigated to determine whether the adsorption is spontaneous and whether each process is exothermic or endothermic; this was done by calculating enthalpy change, Gibbs free energy change, and entropy change values for each process.

#### 3.3.1. Isotherm Models

##### Equilibrium Isotherm Models

Analysis of isotherm data is important since the results can be used to accurately explain the adsorption mechanism. The most common isotherms that are applied in solid/liquid extraction systems are Langmuir and Freundlich isotherms [27].

##### Langmuir Adsorption Isotherm

The Langmuir model represents the chemisorption process. This adsorption is limited to monolayer coverage, in which the adsorbed molecule cannot migrate across the adsorbent surface or interact with neighboring molecules. In addition, the surface of the adsorbent in the Langmuir model is uniform (i.e., adsorption sites all have the same amount of energy) [28]. The Langmuir model studies adsorbate coverage on a solid surface to the concentration of a medium above the adsorbent solid surface at a fixed temperature. The Langmuir model is shown in Equation (3).

(3) $$\frac{C_{e}}{q_{e}}=\frac{1}{bQ_{o}}+\frac{1}{Q_{o}}C_{e}$$

where *C_e_* is the equilibrium concentration of tetraconazole (mg·L^−1^), *b* is a constant that represents the Langmuir affinity (L/mg), *Q_o_* is the adsorption capacity at equilibrium (mg/g), and *q_e_* represents the amount of tetraconazole divided by the adsorbent mass (mg/g).

A graph of (*C_e_*/*q_e_*) versus *C_e_* is used for finding out Langmuir parameters, which are (1/*Q_o_*) as the slope and (1/*bQ_o_*) as the *y* intercept [27,28].

##### Freundlich Adsorption Isotherm

The Freundlich isotherm can be interpreted as adsorption to surfaces supporting sites of different affinities or adsorption on heterogeneous adsorbent surfaces. This isotherm assumes that stronger adsorbent binding sites are occupied at the beginning, in which the binding strength decays with increasing site occupation degree. According to the Freundlich model, the adsorbed mass per mass of adsorbent can be expressed by Equation (4) [29].

(4) $$\log q_{e}=\log K_{F}+\frac{1}{n}\log C_{e}$$

where *K_F_* is a constant for the Freundlich isotherm, which detects the adsorption capacity (mg/g), and *n* represents the heterogeneity coefficient, which is used as an indication to know whether the adsorption process is favorable (g/L).

A plot of log*q_e_* values versus log*C_e_* is used to find Freundlich parameters, which are log*K*_F_ as the *y* intercept and (1/*n*) as the slope.

The adsorption of tetraconazole on the CNC, Cell-F, and Cell-P were fitted to Langmuir and Freundlich isotherms. The adsorption parameters were investigated by plotting *C_e_*/*q_e_* versus *C_e_* for the Langmuir adsorption isotherm and log*q_e_* versus log*C_e_* for the Freundlich adsorption isotherm. All parameters obtained from Figures S3–S5 are summarized in Table 1. The results showed that the adsorption of tetraconazole by the three polymers follows Langmuir adsorption. The *R*^2^ value for the three polymers, CNC, Cell-F, and Cell-P, were 0.9974, 0.9992, and 0.9992, respectively. The values indicate that the adsorption of tetraconazole follows the Langmuir isotherm model.

The *R*^2^ values were close to 1, indicating a high affinity of the polymer to tetraconazole.

#### 3.3.2. Adsorption Kinetic Models

These kinetic models give information about the adsorption system behavior and the rate at which a specific constituent is removed by the adsorbents. In addition, with the kinetic models, whether the adsorption process is chemical or physical can be described, and the rate-determining step can be spotted. Several adsorption kinetic models have been established. Examples of adsorption kinetic models include the external mass transfer model, the first-order reversible reaction model, the pseudo-first-order and pseudo-second-order rate models, the intra-particle-diffusion kinetic model, and others [30,31,32].

##### Pseudo-First-Order Kinetics

The equation for this kinetic model is shown below:

(5) $$\log\left(q_{e}-q_{t}\right)=\log q_{e}-\frac{K_{1}}{2.303}t$$

where *q_e_* and *q_t_* are the mass of the adsorbate per unit mass of the adsorbent at equilibrium at time *t*, respectively (mg/g), and *K*_1_ represents the rate constant for pseudo-first-order kinetic adsorption (mg·g^−1^·min^−1^).

A plot of log (*q_e_* − *q_t_*) versus time will give a straight line for the pseudo-first-order model, in which log *q_e_* is the *y* intercept and (−*K*_1_/2.303) is the graph slope [33].

##### Pseudo-Second-Order Kinetic Model

This adsorption model of kinetics suggests that the rate-determining step can be fitting with chemical adsorption that involves valence forces in which electrons are shared or exchanged between the adsorbent and the adsorbate.

The pseudo-second-order model can be expressed by Equation (6):

(6) $$\frac{t}{q_{t}}=\frac{1}{k_{2}q_{e}^{2}}+\frac{1}{q_{e}}t$$

where *k*_2_ represents the rate constant of pseudo-second-order kinetic adsorption at equilibrium (g·mg^−1^·min^−1^).

The graph of (*t*/*q_t_*) versus time gives a linear relationship that allows the computation of the rate constants in the pseudo-second-order kinetic model, *k*_2_ and *q_e_* [34].

##### Intra-Particle-Diffusion Kinetic Adsorption Model

Intra-particle diffusion assumes a theory that was proposed by the scientists Morris and Weber, and the equation is expressed as follows:

(7) $$q_{t}=K_{p}t^{0.5}+\;C$$

where *K_p_* represents rate constant for the intra-particle-diffusion model (mg/g·min^1/2^) and *C* is a constant related to the boundary layer thickness (mg/g) [35].

A graph of *q_t_* versus *t*^1/2^ will give a linear relationship of the intra-particle-diffusion kinetic model, in which the *C* constant represents the *y* intercept and *K_p_* represents the plot slope [36].

The kinetic experimental data of tetraconazole adsorption on the CNC, Cell-F, and Cell-P were fitted with pseudo-first-order, pseudo-second-order, and intra-particle-diffusion models for investigating the adsorption mechanism of each process.

The kinetics adsorption parameters and correlation coefficients were calculated from the linear plots of log(*q_e_* − *q_t_*) versus time for the pseudo-first-order adsorption model, (*t*/*q_t_*) versus time for the pseudo-second-order model, and *q_t_* versus *t*^1/2^ for the intra-particle-diffusion model. Results are summarized in Figures S6–S8.

According to the correlation coefficient obtained from the kinetic models, the comparison between *q_e_* experimental and calculated values, as shown in Table 2, showed that the adsorption of tetraconazole on all three polymers, the CNC, Cell-F, and Cell-P, follows the pseudo-second-order kinetic model. The results indicate binding between adsorbent and adsorbate.

#### 3.3.3. Adsorption Thermodynamics

Thermodynamic studies of any adsorption process are vital to investigate whether such adsorption is a favorable process. The thermodynamic adsorption behavior is investigated depending on calculating the thermodynamic parameters: the change in Gibbs free energy (∆*G*), the change in entropy (∆*S*), and enthalpy change (∆*H*), in which ∆*G* and ∆*H* have a unit of J, and the unit of ∆*S* is J/K [34,35,36,37].

Equation (8) represents the general relation between the adsorption thermodynamic parameters:

∆*G* = ∆*H* − *T*∆*S* (8)

where *T* represents the absolute temperature (K).

The Gibbs free energy change can also be expressed using Equation (9):

∆*G* = −*RT* In*K_d_* (9)

where *R* represents the universal gas constant with a value equal to 8.314 J·mol^−1^·K and *K_d_* is a thermodynamic equilibrium constant, which equals *q_e_*/*C_e_* with a unit of L/g or mol.

The combination of Equations (8) and (9) will result in the following equation:

(10) $$\ln K_{d}=\frac{\Delta S}{R}-\frac{\Delta H}{RT}$$

A graph of In*K_d_* versus 1/*T* results in a line with a plot slope equal to −∆*H*/*R* and a *y* intercept equal to ∆*S*/*R*. The graph is called the Van’t Hoff plot [37].

The Van’t Hoff plot was used to calculate the thermodynamic parameters (Δ*H*, Δ*G*, and Δ*S* values) for the adsorption of tetraconazole on the CNC, Cell-F, and Cell-P. The plots of In*K*_d_ versus 1/*T* are shown in Figures S9–S11. The values of the obtained thermodynamic parameters Δ*H*, Δ*G*, and Δ*S* are summarized in Table 3.

According to the table above, ∆*G*° and ∆*H* are negative values. This indicates that all adsorption processes of tetraconazole on the CNC, Cell-F, and Cell-P are exothermic (∆*H* < 0) and spontaneous (∆*G* < 0) at the tested temperatures. A negative change in entropy values indicates the presence of an associative mechanism in which no significant change will occur in the internal structure of each adsorbent during the adsorption process.

##### Adsorbents’ Regeneration

**Figure 11:**
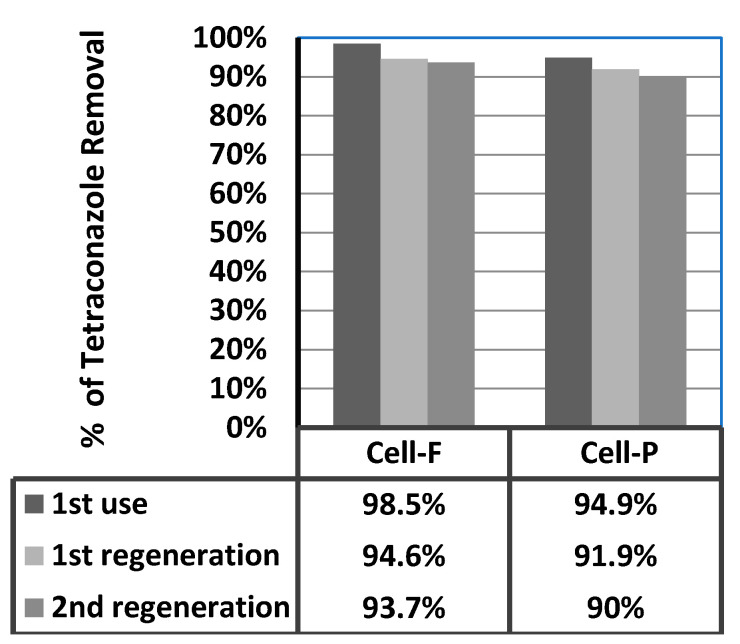
Effect of adsorbent recovery on tetraconazole adsorption on Cell-F and Cell-P (solution volume = 10 mL).

After each run, the adsorbents were rinsed with diluted sulfuric acid (0.1 N), water, and ethanol to ensure complete removal of the adsorbed tetraconazole. Each adsorbent was used for three adsorption cycles, and the results are shown in Figure 11. In the three cycles, a small drop in the adsorption efficiency was noticed. These results indicated that Cell-F and Cell-P could be promising absorbents on a commercial scale for tetraconazole and other pesticides.

## 4. Conclusions

Cellulose-based derivatives with heterocyclic rings were synthesized by reacting cellulose with furan-2-carbonyl chloride (Cell-F) and crosslinking with pyridine-2,6-dicarbonyl dichloride (Cell-P). The prepared adsorbents were characterized by SEM, TGA, IR, and H^1^ NMR instruments. The derivatives were evaluated as adsorbents for tetraconazole from aqueous solution. The optimum adsorption conditions of contact time, pH, temperature, adsorbent dose, and initial concentration of adsorbate were determined. The removal efficiency of tetraconazole from water by Cell-F and Cell-P was 98.5%, and 95%, respectively. Underivatized nanocellulose showed a removal efficiency of about 91.7%. The adsorption process followed the Langmuir model. The kinetics study showed that the adsorption is a pseudo-second-order kinetic model. The obtained values of the thermodynamic parameters indicate a spontaneous adsorption process.

## Figures and Tables

**Table 1:** The parameters of Langmuir and Freundlich isotherms for tetraconazole adsorption on the CNC, Cell-F, and Cell-P.

Polymer	Langmuir Isotherm		Freundlich Isotherm	
	*Q_o_* (mg/g)	*b* (L/mg)	*K*_F_ (mg/g)	*n* (g/L)
CNC	6.481	−3.547	8.955	−4.796
Cell-F	7.604	−6.779	8.956	−7.225
Cell-P	6.935	−4.697	8.904	−5.650

**Table 2:** The parameters of pseudo-first-order, pseudo-second-order, and intra-particle-diffusion kinetic models for the adsorption of tetraconazole on the CNC, Cell-F, and Cell-P.

Polymer	Pseudo-First-Order Kinetics		*q_exp_* (mg/g)	Pseudo-Second-Order Kinetics		Intra-Particle-Diffusion Kinetics	
	*q_e_* (mg/g)	*K*_1_ (mg·g^−1^·min^−1^)		*q_e_* (mg/g)	*K*_2_ (g·mg^−1^·min^−1^)	*C* (mg/g)	*K_p_* (mg·g^−1^·min^−0.5^)
CNC	17.179	7.139*10^−3^	7.112	7.380	0.0711	51.126	0.9449
Cell-F	17.95	-	7.619	7.716	0.238	52.046	0.0013
Cell-P	14.184	-	7.992	8.091	0.141	55.838	−0.0091

**Table 3:** The thermodynamic parameters for tetraconazole adsorption on the CNC, Cell-F, and Cell-P.

Polymer	∆*H* (kJ)	∆*G*° (kJ)	∆*S* (J/K)
CNC	−25.513	−4.693	−69.867
Cell-F	−23.383	−4.792	−62.385
Cell-P	−35.705	−5.549	−101.195

## Supplementary Materials

The following are available online at https://www.mdpi.com/2073-4360/13/3/450/s1, Figure S1: IR Spectra of Cell-F. Figure S2: IR Spectra of CMC. Figure S3: Freundlich (A) and Langmuir (B) plots for tetraconazole adsorption on CNC (pH = 4, temperature = 20 °C, contact time = 30 min, adsorbent dose = 10 mg, solution volume = 10 mL). Figure S4: Freundlich (A) and Langmuir (B) plots for Tetraconazole adsorption on Cellulose Modified with Furan-2-carbonyl chloride (pH = 4, temperature = 20 °C, contact time = 15 min, adsorbent dose = 10 mg, solution volume = 10 mL). Figure S5: Freundlich (A) and Langmuir (B) plots for Tetraconazole adsorption on Cellulose Modified with Pyridine-2,6-dicarbonyl dichloride (pH = 6, temperature = 20 °C, contact time = 20 min, adsorbent dose = 10 mg, volume of solution = 10 mL). Figure S6: Pseudo first-order (A), Pseudo second order (B), and Intraparticle diffusion kinetic model (C) for Tetraconazole adsorption on NanoCellulose (C_I_ = 10 ppm, pH = 7, temperature = 20 °C, adsorbent dose = 10 mg, volume = 10 mL). Figure S7: Pseudo first-order (A), Pseudo second order (B), and Intraparticle diffusion kinetic model (C) for Tetraconazole adsorption on Cellulose Modified with Furan-2-carbonyl chloride (C_I_ = 10 ppm, pH = 7, temperature = 20 °C, adsorbent dose = 10 mg, volume = 10 mL). Figure S8: Pseudo first-order (A), Pseudo second order (B), and Intraparticle diffusion kinetic model (C) for Tetraconazole adsorption on Cellulose Modified with Pyridine-2,6-dicarbonyl dichloride (C_I_ = 10 ppm, pH = 7, temperature = 20 °C, adsorbent dose = 10 mg, volume = 10 mL). Figure S9: Van’t Hoff plot for the adsorption of Tetraconazole adsorption on NanoCellulose (C_I_ = 4 ppm, pH = 4, contact time = 30 min, adsorbent dose = 10 mg, volume = 10 mL). Figure S10: Van’t Hoff plot for Tetraconazole adsorption on Cell-F (C_I_ = 4 ppm, pH = 4, contact time = 15 min, adsorbent dose = 10 mg, volume = 10 mL). Figure S11: Van’t Hoff plot for Tetraconazole adsorption on Cellulose Modified with Pyridine-2,6-dicarbonyl dichloride (C_I_ = 6 ppm, pH = 6, contact time = 20 min, adsorbent dose = 10 mg, volume = 10 mL).
