# Supplementary material for: Novel, Environment-Friendly Cellulose-Based Derivatives for Tetraconazole Removal from Aqueous Solution
Source: Polymers (Basel). 2021 Jan 30;13(3):450. doi: 10.3390/polym13030450 (PMC7866856; doi:10.3390/polym13030450)
Supplement: Supplementary file 1 [file polymers-13-00450-s001.pdf]

Novel Friendly Environment Cellulose Based Derivatives for Tetraconazole Removal from Aqueous Solution

Bayan Khalaf[1], Othman Hamed[1,*], Shehdeh Jodeh[1,*], Erwin Klumpp[2], Ghadir Hanbali[1], Roland Bol[2], Liming Wang[2], Nina Siebers[2]

[1]Department of Chemistry, Faculty of Science, An-Najah National University, P.O. Box 7, Nablus, Palestine.

[2] Institute of Bio and Geosciences, Agrosphere (IBG-3), Forschungszentrum Jülich GmbH, 52425 Jülich, Germany.

[*] Correspondence: sjodeh@hotmail.com, Tel.: +970-599-590-498; ohamed@najah.edu

+970-594-466-271

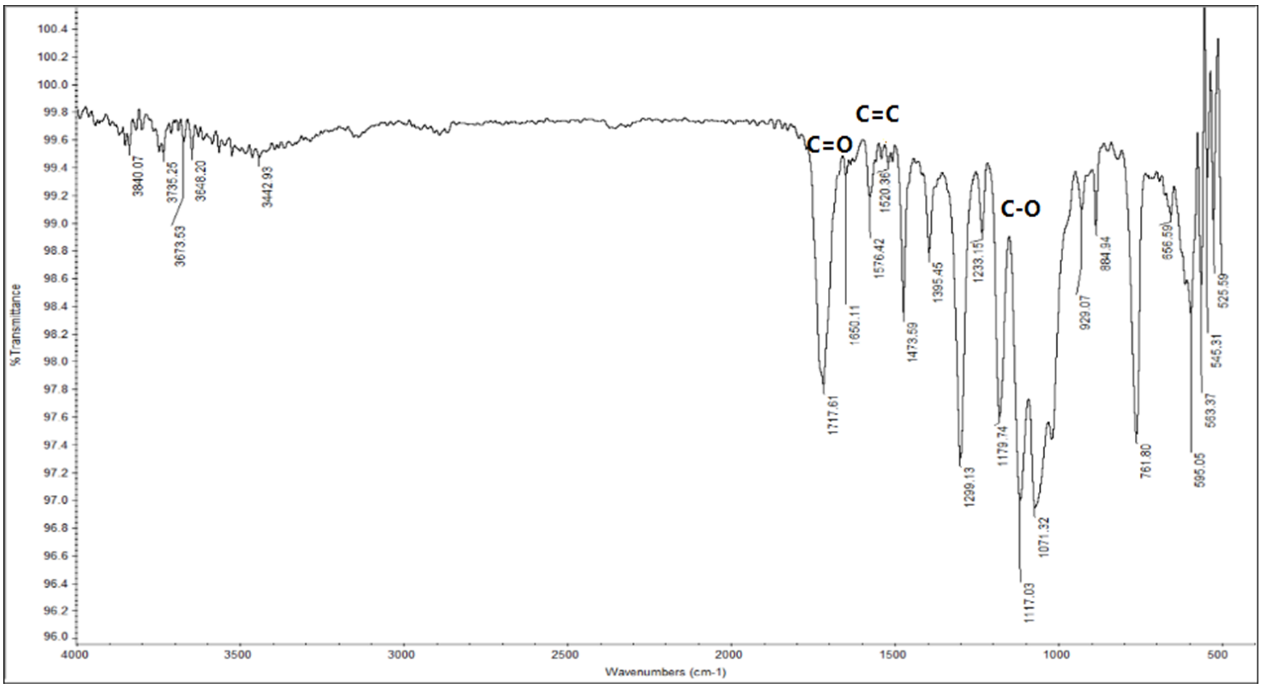

Figure S1: IR Spectra of Cell-F.

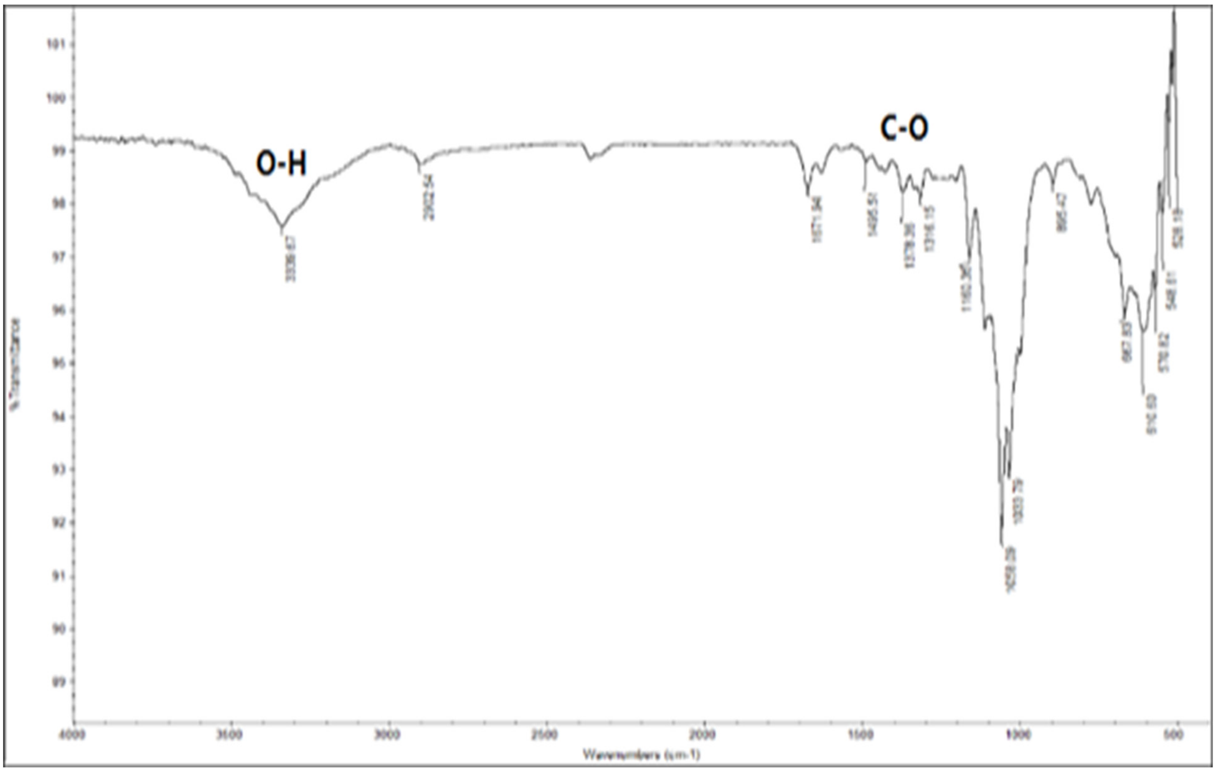

Figure S2: IR Spectra of CMC.

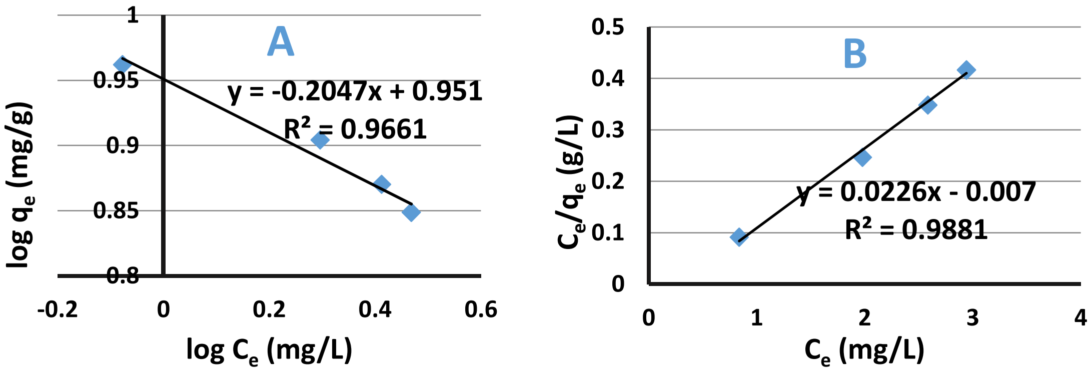

Figure S3: Freundlich (A) and Langmuir (B) plots for tetraconazole adsorption on CNC (pH = 4, temperature = 20 °C, contact time = 30 minute, adsorbent dose = 10 mg, solution volume = 10 mL).

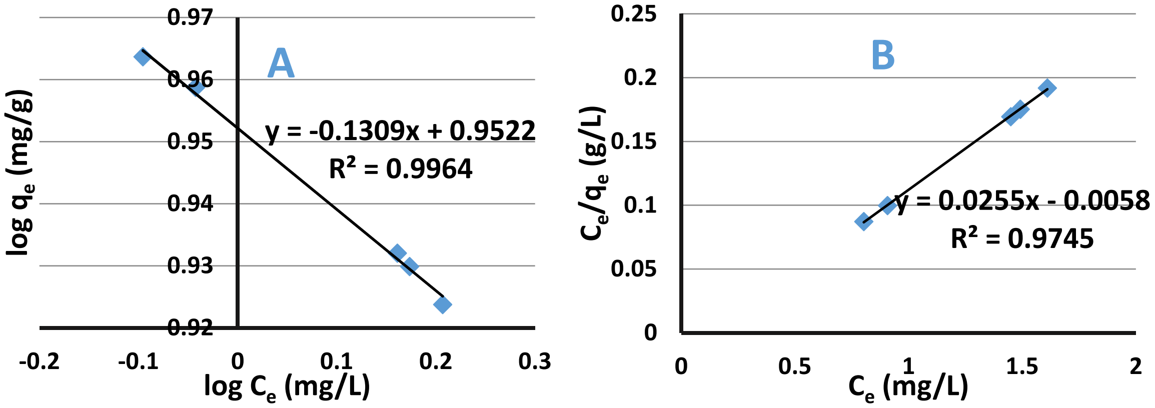

Figure S4: Freundlich (A) and Langmuir (B) plots for Tetraconazole adsorption on Cellulose Modified with Furan-2-carbonyl chloride (pH = 4, temperature = 20 °C, contact time = 15 minute, adsorbent dose = 10 mg, solution volume = 10 mL).

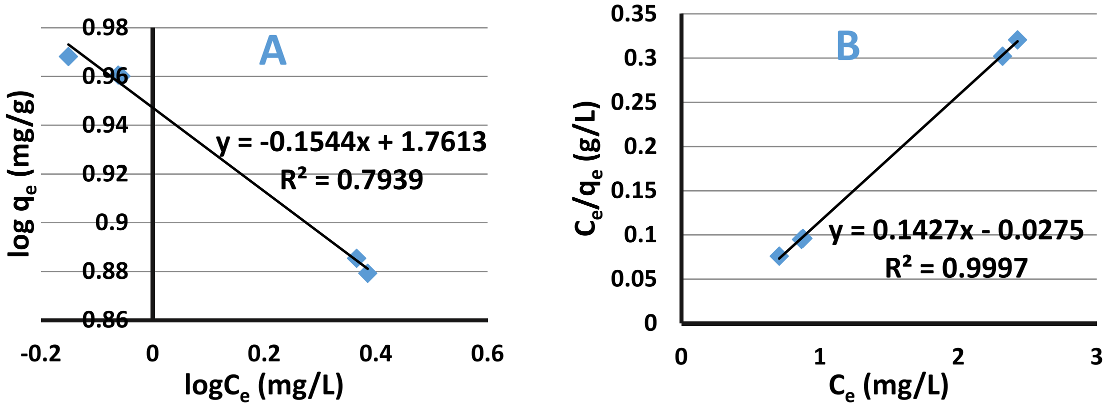

Figure S5: Freundlich (A) and Langmuir (B) plots for Tetraconazole adsorption on Cellulose Modified with Pyridine-2,6-dicarbonyl dichloride (pH = 6, temperature = 20 °C, contact time = 20 minute, adsorbent dose = 10 mg, volume of solution = 10 mL).

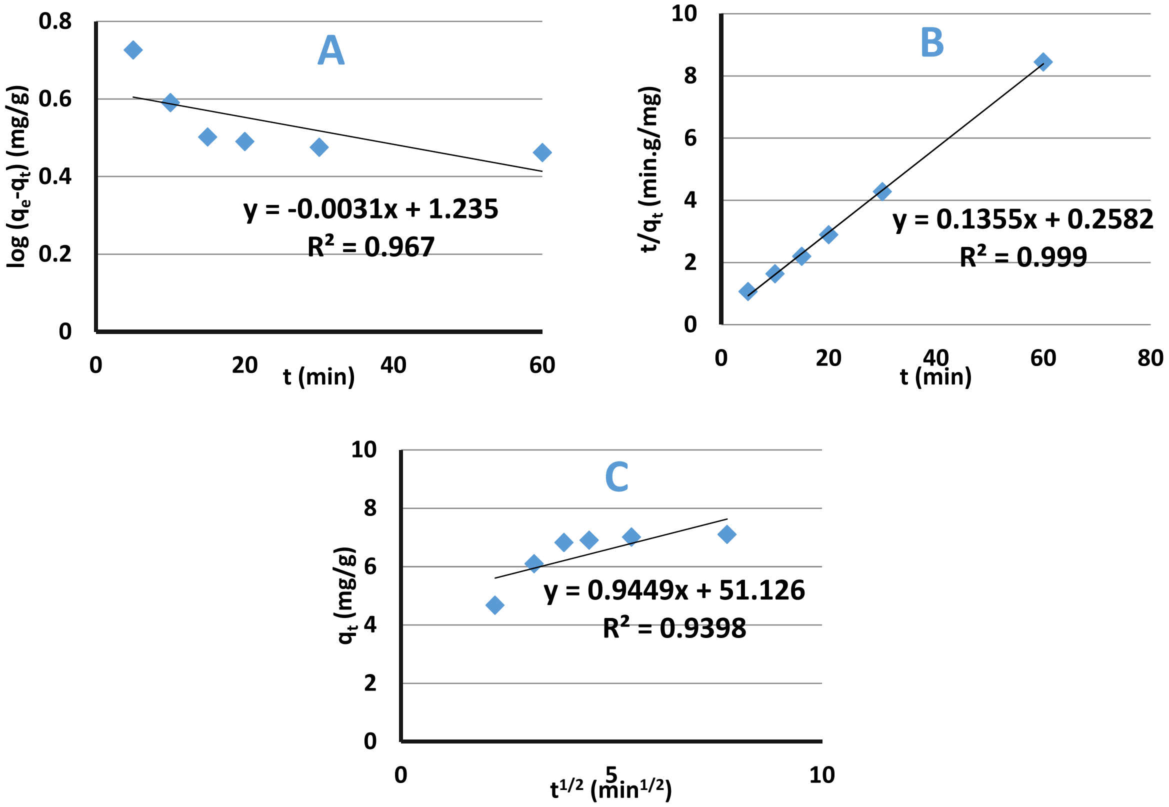

Figure S6: Pseudo first-order (A), Pseudo second order (B), and Intraparticle diffusion kinetic model (C) for Tetraconazole adsorption on NanoCellulose (C_I = 10 ppm, pH = 7, temperature = 20 °C, adsorbent dose = 10 mg, volume = 10 mL).

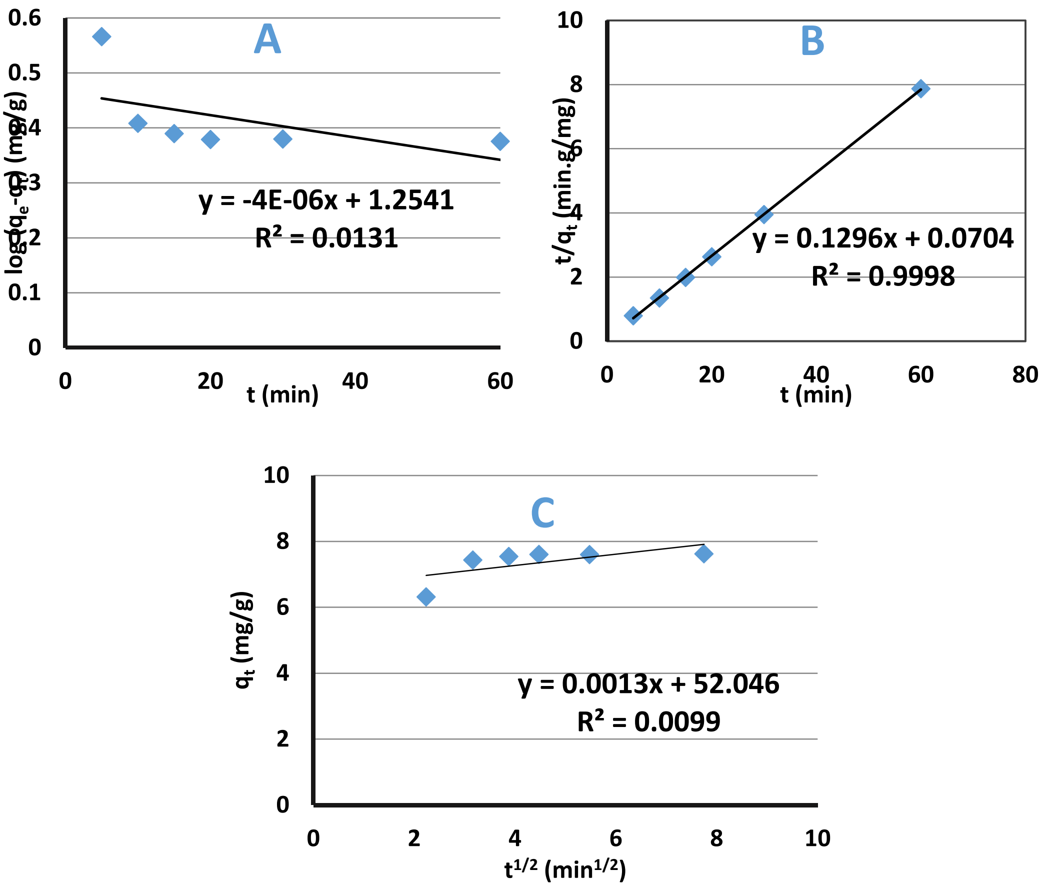

Figure S7: Pseudo first-order (A), Pseudo second order (B), and Intraparticle diffusion kinetic model (C) for Tetraconazole adsorption on Cellulose Modified with Furan-2-carbonyl chloride (C_I = 10 ppm, pH = 7, temperature = 20 °C, adsorbent dose = 10 mg, volume = 10 mL).

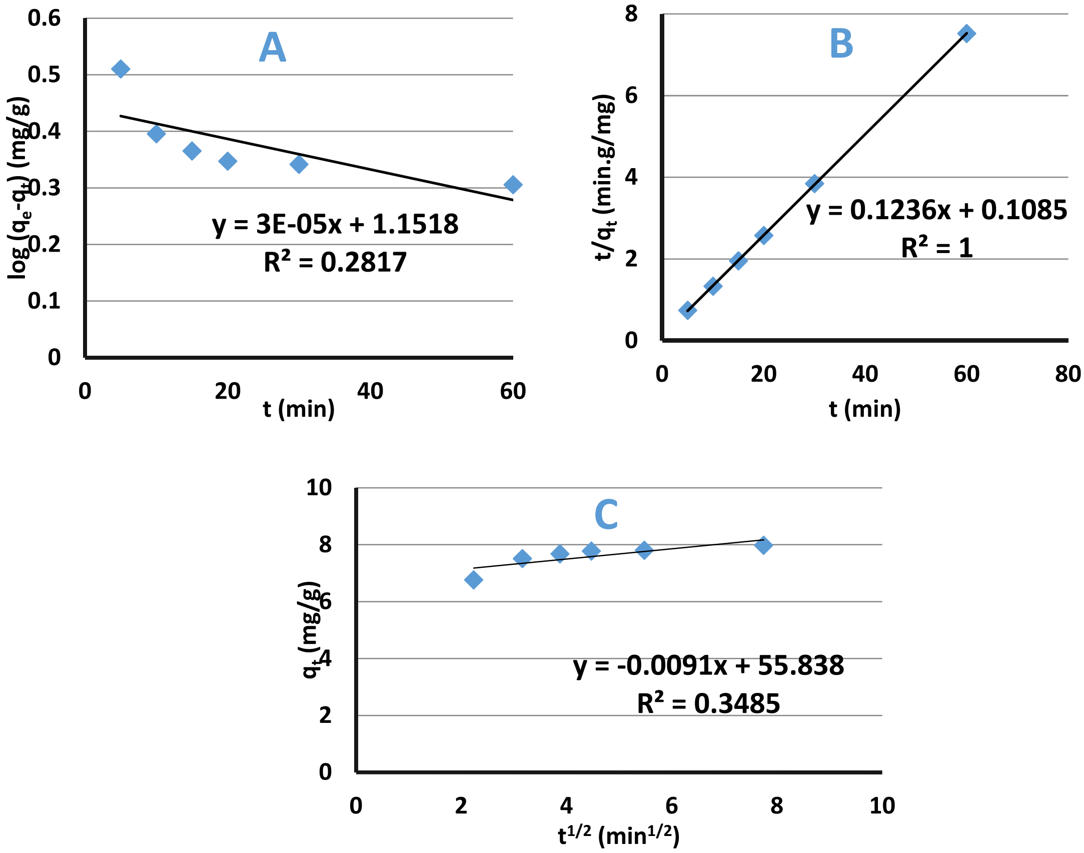

Figure S8: Pseudo first-order (A), Pseudo second order (B), and Intraparticle diffusion kinetic model (C) for Tetraconazole adsorption on Cellulose Modified with Pyridine-2,6-dicarbonyl dichloride (C_I = 10 ppm, pH = 7, temperature = 20 °C, adsorbent dose = 10 mg, volume = 10 mL).

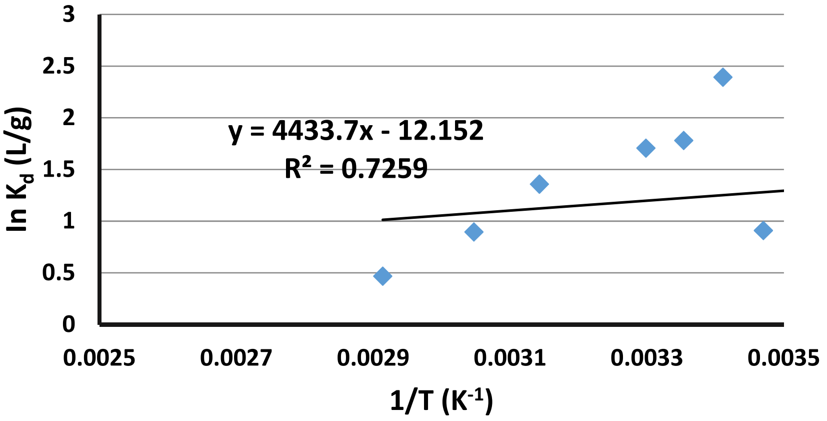

Figure S9: Van't Hoff plot for the adsorption of Tetraconazole adsorption on NanoCellulose (C_I = 4 ppm, pH = 4, contact time = 30 minute, adsorbent dose = 10 mg, volume = 10 mL).

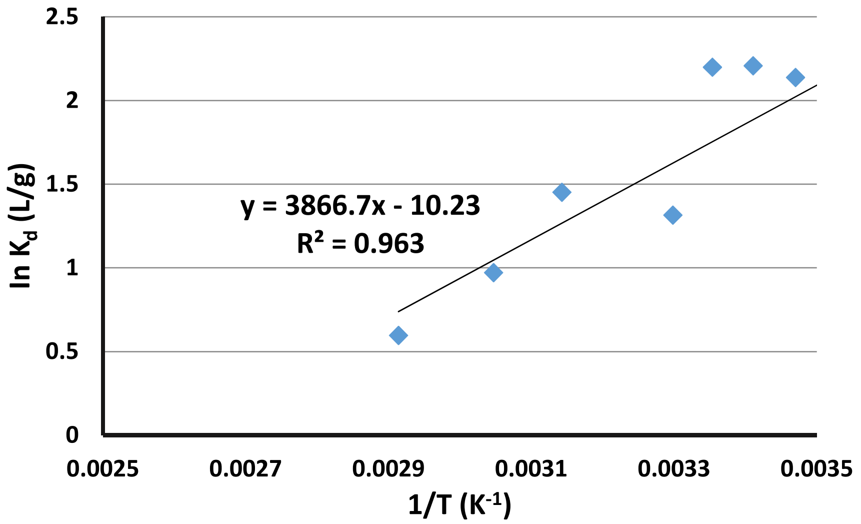

Figure S10: Van't Hoff plot for Tetraconazole adsorption on Cell-F ($C_I = 4$ ppm, pH = 4, contact time = 15 minute, adsorbent dose = 10 mg, volume = 10 mL).

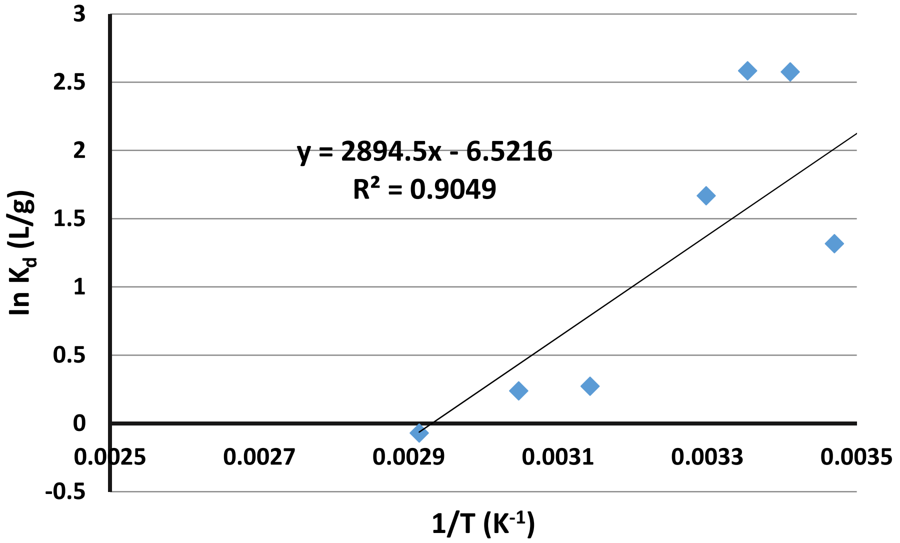

Figure S11: Van't Hoff plot for Tetraconazole adsorption on Cellulose Modified with Pyridine-2,6-dicarbonyl dichloride ($C_I = 6$ ppm, pH = 6, contact time = 20 minute, adsorbent dose = 10 mg, volume = 10 mL).
